# Loss of Hyaluronan and Proteoglycan Link Protein-1 Induces Tumorigenesis in Colorectal Cancer

**DOI:** 10.3389/fonc.2021.754240

**Published:** 2021-12-13

**Authors:** Yao Wang, Xiaoyue Xu, Jacqueline E. Marshall, Muxue Gong, Yang Zhao, Kamal Dua, Philip M. Hansbro, Jincheng Xu, Gang Liu

**Affiliations:** ^1^ College of Biology and Food Engineering, Anyang Institute of Technology, Anyang, China; ^2^ Hangzhou Xunyao Biotechnology Pty. Ltd., Hangzhou, China; ^3^ School of Population Health, University of New South Wales, Sydney, NSW, Australia; ^4^ Centre for Inflammation, Centenary Institute, Sydney, NSW, Australia; ^5^ School of Life Sciences, Faculty of Science, University of Technology, Sydney, NSW, Australia; ^6^ School of Clinical Medicine, Bengbu Medicine College, Bengbu, China; ^7^ Department of Biochemistry and Molecular Biology, School of Medicine, Nanjing University of Chinese Medicine, Nanjing, China; ^8^ Discipline of Pharmacy, Graduate School of Health, University of Technology Sydney, Sydney, NSW, Australia; ^9^ Stomatology Department, The First Affiliated Hospital of Bengbu Medical College, Bengbu, China; ^10^ School of Dental Medicine, Bengbu Medical College, Bengbu, China

**Keywords:** HAPLN1, extracellular matrix protein, microenvironment, TGF-β, CRC

## Abstract

Colorectal cancer (CRC) is the third most common diagnosed cancer worldwide, but there are no effective cures for it. Hyaluronan and proteoglycan link protein-1 (HAPLN1) is a component of the extracellular matrix (ECM) proteins and involved in the tumor environment in the colon. Transforming growth factor (TGF)-β is a key cytokine that regulates the deposition of ECM proteins in CRC. However, the role of HAPLN1 in TGF-β contributions to CRC remains unknown. We found that the mRNA expression of *HAPLN1* was decreased in tumors from CRC patients compared with healthy controls and normal tissue adjacent to the tumor using two existing microarray datasets. This was validated at the protein level by tissue array from CRC patients (*n* = 59). HAPLN1 protein levels were also reduced in human CRC epithelial cells after 24 h of TGF-β stimulation, and its protein expression correlated with type I collagen alpha-1 (COL1A1) in CRC. Transfection of *HAPLN1* overexpression plasmids into these cells increased protein levels but reduced COL1A1 protein, tumor growth, and cancer cell migration. TGF-β stimulation increased Smad2/3, p-Smad2/3, Smad4, and E-adhesion proteins; however, *HAPLN1* overexpression restored these proteins to baseline levels in CRC epithelial cells after TGF-β stimulation. These findings suggest that HAPLN1 regulates the TGF-β signaling pathway to control collagen deposition *via* the TGF-β signaling pathway and mediates E-adhesion to control tumor growth. Thus, treatments that increase HAPLN1 levels may be a novel therapeutic option for CRC.

## Introduction

Colorectal cancer (CRC) is the third most commonly diagnosed cancer worldwide and the second most lethal with more than 900,000 deaths annually (1). The number of new cases is predicted to reach 2.5 million in 2035 (1). With early diagnosis, the 5-year survival rate of CRC is ~65%, but this drops to less than 10% at later stages (2). The cause of tumor formation in CRC remains unknown. Polyps induce long-term inflammation in the colon and may contribute to tumor development of CRC (3). Tissue microenvironment changes also result in tumor formation in CRC. Therefore, it is important to understand the processes that lead to the development and growth of tumors in CRC.

Extracellular matrix (ECM) proteins are a group of macromolecules that provide structural support to cells. Alterations in ECM protein deposition result in aberrant structural changes in tissues, and ECM protein levels need to be balanced to maintain a normal microenvironment in the colon. Hyaluronan and proteoglycan link protein-1 (HAPLN1) is an ECM component that stabilizes other ECM proteins (4). Studies show that a loss of HAPLN1 proteins promotes metastasis of melanoma in patients, and tumor growth is inhibited by reconstitution of HAPLN1 (5). Also, HAPLN1 has important roles in maintaining endothelial permeability, and its loss promotes metastasis *via* blood vessels (6). However, the role of HAPLN1 in CRC has not been widely studied and remains unclear.

Transforming growth factor (TGF)-β is an important cytokine that has major beneficial functions in wound repair, but is also involved in tumor cell survival, invasion, and metastasis in many cancers, including CRC (7). Indeed, previous studies show that increased TGF-β promotes tumor cell proliferation in CRC patients (8, 9). TGF-β is also a central factor in regulating ECM genes and proteins in many diseases (10). Previous studies showed that TGF-β challenge reduced *HAPLN1* mRNA expression in human brain cells after 24 h (11). Another study found amplified *HAPLN1* mRNA levels in CRC patients (*n* = 15) (12), but protein levels have not been assessed in CRC. Links between TGF-β signaling and HAPLN1 in CRC remain unknown.

We hypothesized that HAPLN1 is a key ECM protein that regulates tumor growth and development in CRC. In this study, HAPLN1 mRNA and protein levels were measured in tumor, normal tissue adjacent to tumors, and healthy control tissue in existing microarray datasets and protein tissue arrays. TGF-β challenge reduced HAPLN1 protein levels in human CRC epithelial cells. We also found that decreased HAPLN1 negatively correlated with collagen expression and contributes to tumor development in CRC. *HAPLN1* overexpression restored protein levels in human CRC cells after TGF-β challenge and reduced collagen and tumor cell growth in CRC. Thus, we propose that TGF-β signaling reduces HAPLN1 levels that leads to collagen production and CRC. These data highlight the role of HAPLN1 as an important suppressor of tumor growth in CRC.

## Materials and Methods

### Gene Expression in Human CRC Microarray Datasets

*HAPLN1* gene expression in colorectal tumor and normal tissue adjacent to tumors from CRC patients and healthy controls was assessed in two existing microarray datasets (GSE128449 and GSE110224) in the Gene Expression Omnibus (GEO). Normal tissues adjacent to tumors were described to be those normal samples dissected from adjacent tumors (>4 cm from the tumor) from the same CRC patients (13). The data were analyzed using Bioconductor in R as previously described (14–16).

In the GSE128449 dataset, gene microarray from colorectal tissues was obtained from healthy controls (*n* = 5) and CRC patients (*n* = 31). Data were profiled by Agilent-014850 Whole Human Genome Microarray 4x44K G4112F. In the GSE110224 dataset, gene microarray from colorectal tissues and normal tissues adjacent to tumors was obtained from 17 CRC patients of similar age during surgery (17). mRNAs were profiled by Affymetrix Human Genome U133 Plus 2.0 array. The Benjamini–Hochberg method for adjusted *P*-value/false discovery rate (FDR) was used to analyze differences between groups. Statistical significance was set at FDR <0.05. All target gene expression was calculated as log_2_ intensity robust multi-array average signals (log_2_-transformed intensity value) (18).

### Human Subjects

Human colon cancer tumor (CD4, SuperBioChips Laboratories, Seoul, South Korea) and normal tissues adjacent to tumor samples (CDN4, SuperBioChips Laboratories, Seoul, South Korea) were obtained from 59 stage I–IV CRC patients in tissue array slides (**Table 1**). The tumor and normal tissues adjacent to tumor tissues were collected during colorectal surgery and fixed with formalin. The tissues were dehydrated with gradient ethanol (70%, 90%, 95%, 99%, and 100%) for 1 h for each progressive step and paraffin embedded at 60°C for 3 h. The tissue blocks were sectioned in 4 μm thickness on new silane III slides (Cat # 5116-20F, Muto Pure Chemicals).

**Table 1:** Patient characteristics (*n* = 59).

	No. of patients	%
Sex		
Male	15	25.5
Female	44	74.5
Age mean = 60.6 ± SD 10.58 (range 35–86 years old)		
Stage		
I	2	3.3
II	33	55.9
III	20	33.9
IV	4	6.9

### Survival Analysis

CRC samples with *HAPLN1* low (*n* = 176) and high (*n* = 242) gene expression were obtained from the colons of CRC patients (stages I–IV, **Supplementary Table S1**) based on The Cancer Genome Atlas Program (TCGA, https://ww.cancer.gov/tcga) (19). The Kaplan–Meier survival curve was produced using R packages survival and survminer by OncoLnc (http://www.oncolnc.org) (20). The relationship between *HAPLN1* gene expression and overall patient survival time was verified using a log-rank test.

### Single-Cell Analysis of Human CRC Dataset

*HAPLN1* gene expression from different cell clusters in CRC was assessed in previously published single-cell RNA-sequencing dataset (21). Single cells were obtained from tumor tissues that were resected from CRC patients (*n* = 31) as previously described (21). RNA-sequencing was performed using a Chromium system (10x Genomics) across eight lanes on a HiSeq 4000 platform. Cells were clustered and visualized using *t*-distributed stochastic neighbor embedding (tSNE) plots as previously described (16, 21). The cellular sources of *HAPLN1* mRNA in CRC were identified using the UCSC cell browser.

### Immunohistochemistry

Slides of human colon cancer and normal tissues adjacent to tumor tissue were incubated at 60°C for 30 min, deparaffinized with xylene (5 min twice), and dehydrated in 100%, 95%, 90%, and 75% ethanol for 2 min (twice each). They were then incubated in citrate buffer (pH 6.0) at 100°C (35 min) for antigen retrieval and blocked with 5% bovine serum albumin (A9418, Sigma-Aldrich) at room temperature for 1 h. Slides were then incubated with HAPLN1 (1:100, sc-46826, Santa Cruz Biotechnology), p-Smad2/3 (1:100, SAB454208, Merck), and type I collagen alpha-1 (COL1A1, 1:100, ab21286, Abcam) antibodies at 4°C overnight, and then with anti-rabbit horseradish peroxidase-conjugated secondary antibody (HAF008, R&D Systems, USA) at room temperature for 1 h. They were washed in PBS-Tween 20 (three times, 5 min) with shaking, and sections were incubated with 3,3′-diaminobenzidine (DAB) chromogen solution at room temperature for 10 min according to the instruction of the manufacturer (GV825, Agilent). Slides were counterstained with hematoxylin at room temperature for 5 min. Images were taken under ×5 and ×20 magnifications using a light microscope. The area of HAPLN1 proteins was calculated using ImageJ with a color deconvolution plug-in (ImageJ) as previously described (22). The percentage of HAPLN1 protein in each image (at least 20 random images) was calculated by the area of positive staining divided by the total area of the view under ×20 magnification.

### Cell Culture

Human CRC epithelial cells (Caco-2, HTB-37, ATCC, Manassas, VA, USA) were maintained in Eagle’s minimum essential medium (EMEM) containing 2.5 mM L-glutamine, 10 mM HEPES, and 10% fetal bovine serum (FBS) at 37°C with 5% CO_2_. Cells (1 × 10^5^ cells/well) were seeded in a six-well plate and cultured in EMEM medium with 0.1% FBS at 37°C with 5% CO_2_ for 24 h. Human recombinant TGF-β protein (50 μg/ml, 7666-MB-005/CF, R&D Systems) was added to the cell media, and control cells received an equal volume of EMEM medium. Cell lysates were collected after 6, 12, 24, and 48 h for immunoblot. Some cells were seeded on coverslips in a 24-well plate overnight and treated with TGF-β recombinant protein for 6, 12, 24, and 48 h, and cells were collected for immunofluorescence assays.

### Protein Extraction

Cell lysates were obtained using radioimmunoprecipitation assay buffer (RIPA; Sigma-Aldrich, St. Louis, USA) supplemented with protease and phosphatase inhibitors (Thermo Fisher Scientific, MA, USA). Lysed cell samples were centrifuged (8,000×*g*, 10 min, 4°C) and supernatants were collected for protein assays. Total protein concentrations in cell lysates were determined using a Pierce bicinchoninic acid (BCA) protein assay kit (Thermo Fisher Scientific, MA, USA) according to the instructions of the manufacturer.

### Immunoblot

Proteins from cell lysates were separated by electrophoresis under 110 V for 1.5 h and transferred onto polyvinylidene difluoride (PVDF) membranes. Membranes were blocked with 5% BSA for 2 h at room temperature, and then incubated with anti-HAPLN1 (1:100, sc-46826, Santa Cruz Biotechnology), COL1A1 (1:2,000, ab21286, Abcam), E-cadherin (1:2,000, 3195S, Cell Signaling), Smad2/3 (1:2,000, 8685S, Cell Signaling), Smad4 (1:2,000, 46535S, Cell Signaling), p-Smad2/3 (1:1,000, SAB454208, Merck), and anti-β-actin (1:10,000, ab8226, Abcam, Cambridge, UK) antibodies at 4°C overnight. Blots were washed with TBS-Tween 20 (three times, 10 min) and incubated with anti-rabbit or anti-mouse IgG HRP-conjugated antibodies (R&D Systems, MN, USA) at room temperature for 2 h. Substrate (SuperSignal™ West Femto Maximum Sensitivity Substrate, Thermo Fisher Scientific, MA, USA) was added to the membrane and images of immunoblots were captured using a ChemiDoc MP System (Bio-Rad, Hercules, USA). Some blots were stripped (15 g glycine, 1 g SDS, 10 ml Tween 20, pH 2.2) but only once to avoid background effects. Densitometry analysis was performed relative to the housekeeping protein β-actin using ImageJ (NIH, Bethesda, USA) as previously described (23, 24). The fold change of normalized area in each challenge/treatment group was compared with the control group.

### Immunofluorescence

Caco-2 cells were fixed with cold methanol (−20°C) and 3% paraformaldehyde in PBS (pH 7.4) both at room temperature for 10 min each, permeabilized with 0.2% Triton X-100, and blocked with 5% BSA at room temperature for 1 h. Cells were incubated with anti-human HAPLN1 (1:100, sc-46826, Santa Cruz Biotechnology), COL1A1 (1:100, ab21286, Abcam), or p-Smad2/3 (1:100, SAB454208, Merck) antibodies at 4°C overnight. After three washes with PBS-Tween 20, cells were incubated with FITC-conjugated anti-rabbit secondary antibody (1:100, ab6717, Abcam) or AF594 anti-rabbit secondary antibody (1:200, ab150080, Abcam) at room temperature for 1 h. Nuclei were counterstained with DAPI at room temperature for 5 min. Ten random images per section were visualized using an Axio Imager M2 microscope and analyzed using an imaging software (Zen, Zeiss) as previously described (24, 25). The percentage of HAPLN1-positive cells was calculated as the percentage of the total cell number (DAPI-positive cells).

### *HAPLN1* Overexpression Treatment

*HAPLN1* overexpression plasmids within a pCMV3-C-GFPSpark^®^ vector (HG10323-ACG, Sino Biological, China) or negative control (CV026, Sino Biological, China) plasmids (200 pg/μl) were transformed into SIG10 chemically competent cells (CMC0001, Sigma-Aldrich) according to the instructions of the manufacturer. Cells were cultured in LB broth at 37°C for 3 h with 200 rpm shaking. Cells were then cultured on LB agar plates (L3147, Sigma‐Aldrich, St. Louis, USA) containing kanamycin sulfate (200 μg/ml, 60615, Sigma‐Aldrich, St. Louis, USA) at 37°C overnight. A single colony was collected and cultured in LB broth at 37°C for 6 h with 200 rpm shaking, and bacterial cells were collected by centrifugation (6,000 rpm at room temperature for 10 min). Plasmids were isolated and purified using a PureLink HiPure Plasmid Midiprep kit (Thermo Fisher Scientific, Waltham, USA) according to the instructions of the manufacturer (25). Caco-2 cells (2 × 10^5^ cells/well) were seeded in EMEM media (0.1% BSA and without antibiotics) on a 24-well plate at 37°C overnight. *HAPLN1* or negative control plasmids (1,000 ng) were transfected into Caco-2 cells using Lipofectamine 3000 transfection reagent (L3000008, Thermo Fisher Scientific, Waltham, USA) according to the instructions of the manufacturer. Cell lysates were collected after 12, 24, and 48 h for immunoblot analysis. Some cells were seeded on coverslips for immunofluorescence assays.

### Cell Proliferation Assay

To investigate cell proliferation, Caco-2 cells (1 × 10^4^ cells/well) were seeded in a 96-well plate and then transfected with *HAPLN1* overexpression or negative control plasmids. Cells were incubated with TGF-β recombinant protein (50 μg/ml) for 24 h and control cells received media only. Cells were stained with crystal violet (10%) at room temperature for 20 min and incubated with methanol at room temperature for 10 min. At least 10 random images were taken using a light microscope under ×40 magnification. Crystal violet-positive cells were enumerated using ImageJ as previously described (26).

### Wound Healing Assay

Caco-2 cells were cultured on a 24-well plate with serum-free media for 24 h. The cells were scratched with a p200 pipette tip to generate a straight line across the center of each well as previously described (26), and then gently washed three times with PBS. Fresh serum-free EMEM media with TGF-β recombinant protein (50 μg/ml) were added to each well and control cells received media only. Cells were incubated at 37°C for 24 h with 5% CO_2_. Images were taken using a phase-contrast microscope at 0, 6, 12, and 24 h after scratching. The area of each scratch at each time point was compared to images from 0 h using ImageJ software.

### Cell Migration Assay

CRC cell migration was assessed using Boyden’s chamber assay as previously described (27). Caco-2 cells (2 × 10^4^ cells/ml) were seeded in upper chambers (Transwell) with 200 μl of DMEM media (1% FBS). Chambers were cultured in wells of 24-well plates containing 750 μl of DMEM overnight. Some cells were transfected with *HAPLN1* overexpression or negative control plasmids for 24 h using Lipofectamine 3000. Controls received equal volumes of media. Cells were then incubated with recombinant TGF-β protein for 24 h. Non-migrated cells remaining on the top surface of Transwell membrane were removed using cotton swabs. Cells that migrated to the lower surface of the membranes were fixed with 10% formalin (15 min) and permeabilized with methanol (5 min). Cells were then stained with crystal violet. Migrated cells were visualized and counted in at least five random fields per well under ×20 magnification using an inverted microscope.

### Tissue Atlas Protein Analysis

Representative immunohistochemistry images of Smad2, Smad3, Smad4, and COL1A1 proteins in human healthy control colon and tumors from CRC patients were obtained from the Human Atlas database (version 19.3) as previously described (16).

### Statistical Analysis

Results are presented as mean ± standard error of the mean (SEM). Each *in-vitro* experiment was performed in triplicate and repeated in three to four independent experiments. Unpaired Student’s *t*-tests were used to compare two groups, and one-way analysis of variance (ANOVA) with Bonferroni comparisons was used to compare the results of more than two groups. All statistical analyses were performed using GraphPad Prism Software (San Diego, CA, USA). Statistical differences were accepted at *P <*0.05.

## Results

### *HAPLN1* Gene Is Decreased in CRC Patients

**Figure 1:**
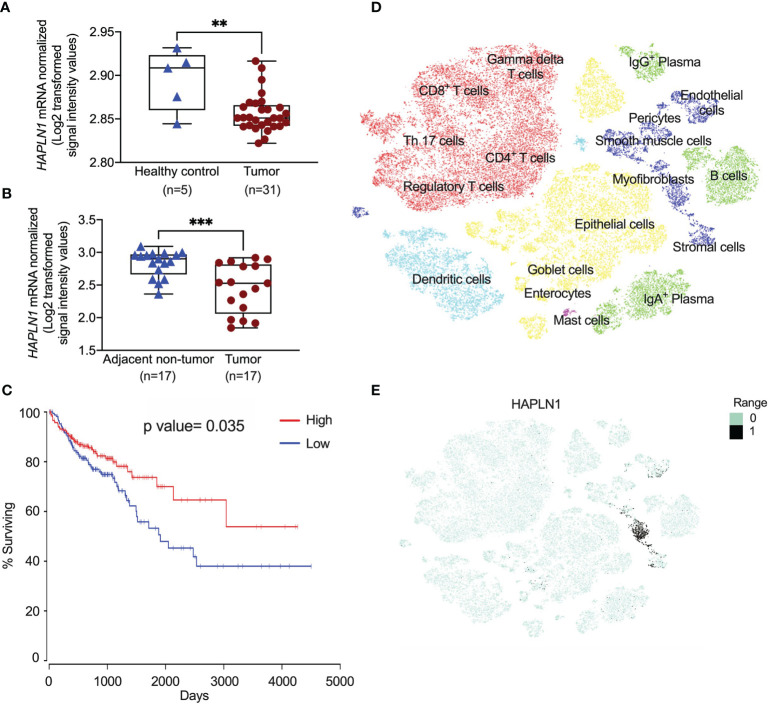
*HAPLN1* gene expression is decreased in colorectal cancer (CRC) patients. **(A)** *HAPLN1* gene expression was analyzed in colon tissues from healthy (*n* = 5) and CRC patients (*n* = 31) in the GSE128449 dataset. **(B)** *HAPLN1* gene expression was analyzed in colon tissues from tumor and normal tissue adjacent to the tumor from the same CRC patients (*n* = 17) in the GSE110224 dataset. **(C)** The Kaplan–Meier survival curve of the correlations between the mRNA expression of the *HAPLN1* gene and survival days, and the cutoff value of RNA expression was 27.8. **(D)** Single cells were isolated from tumors in CRC patients (*n* = 31) after surgery. Cells were clustered using a graph-based shared nearest neighbor clustering approach and visualized in a *t*-distributed stochastic neighbor embedding (tSNE) plot in UCSC cell browser. **(E)** mRNA expression of *HAPLN1* in different cells in CRC patients. Results are mean ± SEM. **P < 0.01, ***P < 0.001 compared with healthy control or non-tumor adjacent tissues.

Previous studies showed that HAPLN1 was involved in tissue remodeling (6), but its role in CRC remains unknown. We assessed the *HAPLN1* mRNA levels in CRC patients and colons from healthy controls using an existing microarray dataset (GSE128449). We found that *HAPLN1* mRNA was significantly decreased in CRC patients compared with healthy tissues (**Figure 1A**). To further assess the level of *HAPLN1* in CRC patients, we measured *HAPLN1* mRNA in tumor tissues and their normal tissues adjacent to the tumor in CRC patients using another microarray dataset (GSE110224). *HAPLN1* mRNA levels were also decreased in tumor compared with normal tissues adjacent to tumor tissues in the same CRC patients (**Figure 1B**). We also found that low expression of the *HAPLN1* gene in CRC patients correlated with shorter survival periods than those with high *HAPLN1* expression (*P* = 0.035, **Figure 1C**). To define the cellular source of HAPLN1 in CRC, tumors were collected from 31 CRC patients (21) and single-cell RNA-sequencing analysis was preformed (**Figure 1D**). *HAPLN1* mRNA was found in epithelial, stromal, and endothelial cells and myofibroblasts (**Figure 1E**).

### Decreased HAPLN1 Protein in CRC Patients

**Figure 2:**
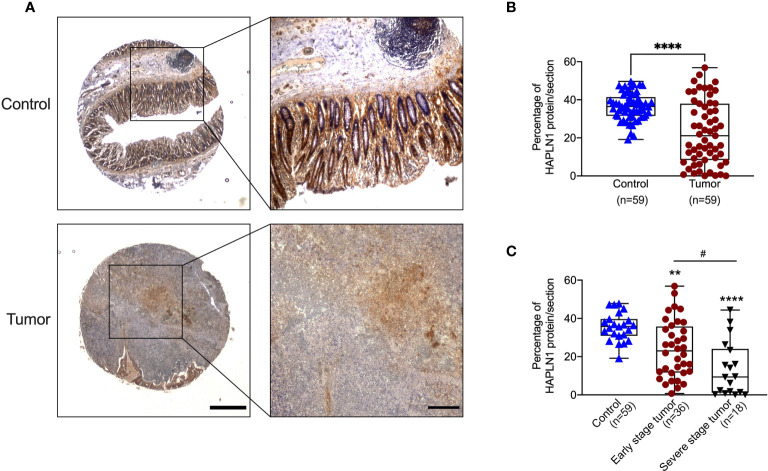
Colorectal cancer (CRC) patients have decreased HAPLN1 protein in tumors. **(A)** Colon tissues were collected from tumors and normal tissue adjacent to the tumor from CRC patients (*n* = 59). Tissues were sectioned and stained with HAPLN1 antibody by immunohistochemistry (low magnification scale bar = 200 μm; high magnification scale bar = 50 μm). **(B)** The area of HAPLN1 staining in all stages of CRC patients was normalized to the total area of the colon section. **(C)** The area of HAPLN1 staining in early (stages I and II, *n* = 36) and severe stages (stages III and IV, *n* = 18) of CRC patients was normalized to the total area of the colon section. Results are mean ± SEM. **P < 0.01, ****P < 0.0001 compared with a normal tissue adjacent to the tumors from CPC patients. ^#^ *P* < 0.05 compared with early stage tumour of CPC patients.

To confirm that the decrease of *HAPLN1* mRNA translates to reduced protein levels in CRC patients, we assessed the HAPLN1 protein in tumor and normal tissues adjacent to tumor tissues from 59 CRC patients using immunohistochemistry (**Figure 2A**), and the HAPLN1 protein was mainly produced by CRC epithelial cells. The HAPLN1 protein was decreased in tumor (stage I–IV cancer) compared with normal tissues adjacent to tumor tissues (mean = 35.56 vs. 22.73, **Figure 2B**). The level of HAPLN1 protein was further reduced in severe stage (III–IV) CRC compared with early stages (mean 13.48 vs. 10.5, **Figure 2C**).

### TGF-β Reduces HAPLN1 Proteins in Cancer Cells

**Figure 3:**
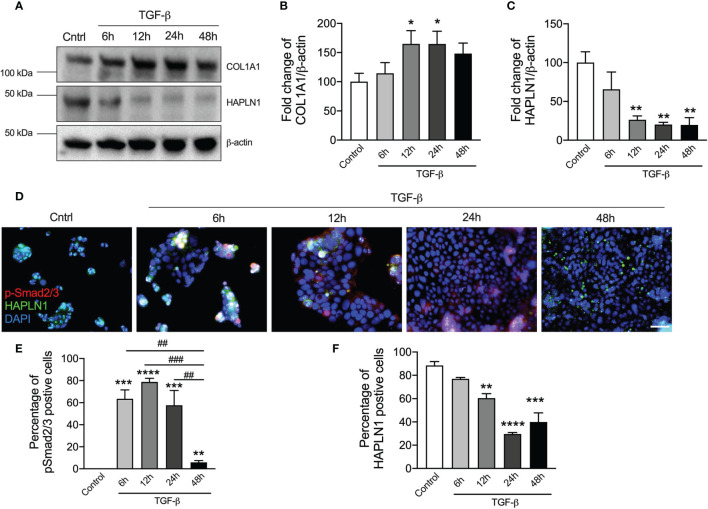
TGF-β stimulation reduces HAPLN1 protein in human colorectal cancer (CRC) cells. Human CRC epithelial cells (Caco-2) were challenged with recombinant TGF-β protein (5 ng/μl), and controls cells received media. **(A)** HAPLN1 and COL1A1 protein levels were assessed in cell lysates over a time course (6, 12, 24, and 48 h) of TGF-β challenge by immunoblot. Fold change of densitometry of HAPLN1 **(B)** and COL1A1 **(C)** was normalized to β-actin. **(D)** Caco-2 cells were stained with HAPLN1 and nuclei were stained with DAPI and assessed using immunofluorescence (*n* = 4, scale bar = 100 μm). p-Smad2/3 **(E)** and HAPLN1 **(F)**-positive cells were normalized to total cells to determine the percentage of HAPLN1-positive cells. Results are mean ± SEM. *P < 0.05, **P < 0.01, ***P < 0.001, ****P < 0.0001 compared with control cells that received media. ^##^ *P* < 0.01, ^###^ *P* < 0.001 compared with TGF-β-challenged cells after 48 h.

Many studies have shown that CRC is associated with increased TGF-β protein and its downstream signaling is the key driver of tumor development (28). Previous studies also showed that TGF-β-induced ECM proteins led to the changes in the tissue microenvironment and tumor formation (26). COL1A1 is the most abundant ECM proteins in human (10). We examined the presence of abnormal ECM deposition and microenvironment change in CRC using TGF-β challenge of human CRC epithelial cells and assessing the levels of COL1A1 proteins over a time course (6, 12, 24, and 48 h, **Figure 3A** and **Supplementary Figure S1A**). TGF-β challenge resulted in significantly increased COL1A1 protein in the cell lysates reaching a peak after 24 h (**Figure 3B**).

To assess the role of HAPLN1 in CRC, we then measured its protein levels in cell lysates by immunoblot (**Figure 3A**). TGF-β challenge reduced HAPLN1 proteins in CRC epithelial cells from the 6-h time point with the maximal decrease after 24 h compared with controls (**Figure 3C**).

To further assess HAPLN1 in CRC, we challenged human CRC epithelial cells with TGF-β and assessed downstream signaling and HAPLN1 by immunofluorescence (**Figure 3D**). p-Smad2/3 (active version of Smad2/3) is an important downstream mediator in the TGF-β signaling pathway. p-Smad2/3-positive cells increased after 6 h of TGF-β compared with controls, were then maintained at the same level up to 24 h, but then decreased at further time points (48 h, **Figure 3E**). TGF-β reduced the percentage of HAPLN1-positive cells, with the greatest effect after 24 h (**Figure 3F**), and this confirmed our result in cell lysates.

### Overexpression of *HAPLN1* Gene in CRC Epithelial Cells Reduces Tumor Growth

**Figure 4:**
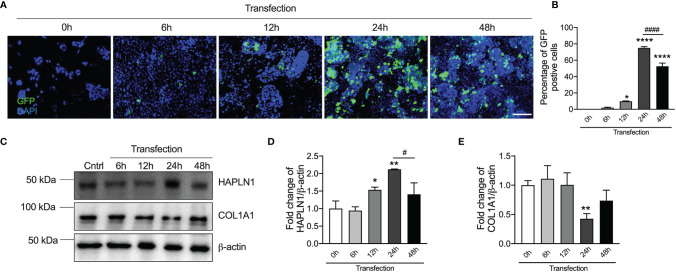
*HAPLN1* overexpression increases HAPLN1 but decreases COL1A1 proteins in human colorectal cancer (CRC) epithelial cells. *HAPLN1* overexpression plasmids conjugated with GFP were transfected into human CRC epithelial (Caco-2) cells. **(A)** GFP-positive cells were assessed over a time course of transfection (0, 6, 12, 24, and 48 h) by immunofluorescence (*n* = 4, scale bar = 100 μm), and **(B)** GFP-positive cells were normalized to total cells (per view). **(C)** HAPLN1 and COL1A1 proteins were assessed in cell lysates over a time course of *HAPLN1* overexpression plasmid transfection (0, 6, 12, 24, and 48 h) by immunoblot. Fold changes of densitometry of HAPLN1 **(D)** and COL1A1 **(E)** were normalized to β-actin. *n* = 4. Results are mean ± SEM. *P < 0.05, **P < 0.01, ****P < 0.0001 compared with cells 0 h after HAPLN1 overexpression plasmid transfection. ^#^ *P* < 0.05, ^####^ *P* < 0.0001 compared with cells 48 h after *HAPLN1* overexpression plasmid transfection.

Given that CRC development was associated with a decrease in HAPLN1 protein levels, to further understand its role, we transfected *HAPLN1* overexpression or negative control plasmid into human CRC epithelial cells. We assessed *HAPLN1* overexpression plasmid transfection efficiency by enumerating GFP-positive cells at different time points (6, 12, 24, and 48 h) after plasmid transfection as the plasmid contained the *GFP* gene (**Figure 4A**). The number of GFP-positive cells peaked 24 h after transection (**Figure 4B**) with a decrease after 48 h. We also measured HAPLN1 proteins in cell lysates during the transfection time course by immunoblot (**Figure 4C** and **Supplementary Figure S1B**). HAPLN1 proteins were significantly increased 24 h after transfection, but the levels decreased thereafter (**Figure 4D**), which confirmed the immunofluorescence data (**Figure 4A**). Therefore, we chose 24 h as the optimized transfection time point.

We also found that COL1A1 proteins were decreased in CRC epithelial cells after *HAPLN1* overexpression plasmid transfection (**Figure 4E**). COL1A1 proteins were the lowest after 24 h corresponding with the highest level of HAPLN1 proteins, indicating a correlation between HAPLN1 and COL1A1 proteins.

### HAPLN1 Changes the Tumor Microenvironment by Regulating Collagen Production in CRC

**Figure 5:**
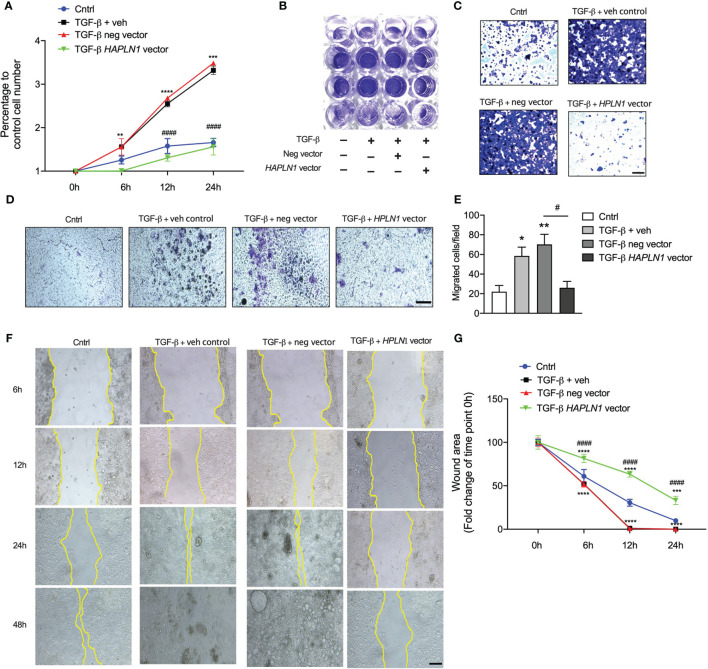
*HAPLN1* overexpression reduces tumor cell growth in colorectal cancer (CRC). Human CRC epithelial (Caco-2) cells were transfected with *HAPLN1* overexpression or negative control plasmid, and vehicle controls received media. Cells were then challenged with human recombinant TGF-β protein. **(A)** Live cells were enumerated during the time course of TGF-β challenge (0, 6, 12, and 24 h) using a cell viability assay. **(B)** Cells were stained with crystal violet after 24 h of TGF-β challenge and **(C)** visualized using light microscopy (scale bar = 100 um). **(D)** Cell migration was assessed by boyden’s chamber assay after 24 hours TGF-β challenge that Caco-2 cells were stained with crystal violet (scale bar = 100 um) and **(E)** migrated were counted in at least 5 random fields per well under 20x magnification lens by an inverted microscope. **(F)** Cell Scratch assays were performed in wells cultured with Caco-2 cells, and cancer cell invasion and migration were assessed by wound healing (scale bar = 200 um). **(G)** Wound area was assessed by measuring wound closure size after 0, 6, 12, and 24 h post-scratch. *n* = 4. Results are mean ± SEM. *P < 0.05, **P < 0.01, ***P < 0.001, ****P < 0.0001 compared with control cells. ^#^ *P* < 0.05, ^####^ *P* < 0.0001 compared with cells transfected with negative control plasmid.

To better understand the role of HAPLN1 in regulating CRC growth, we transfected *HAPLN1* overexpression plasmid with and without TGF-β recombinant protein and examined the effects after 24 h. Human CRC epithelial cell growth significantly increased with concomitant TGF-β protein challenge, with an ~3-fold increase of cells after 24 h compared with controls (**Figure 5A**). *HAPLN1* overexpression reduced CRC growth compared with cells transfected with the negative control plasmid and media controls with concomitant TGF-β (**Figures 5A–C**). We further assessed the role of HAPLN1 in CRC using migration assays (**Figure 5D**). TGF-β recombinant protein significantly increased CRC epithelial cell migration; however, *HAPLN1* overexpression substantially reduced the number of migrated cells (**Figure 5E**). We then assessed the role of HAPLN1 in CRC growth using wound healing assays (**Figure 5F**). The wounds in the CRC epithelial cells started to recover 6 h after TGF-β challenge and resolved after 24 h in cells treated with media control or negative control plasmid (**Figure 5G**). However, cells overexpressing *HAPLN1* had reduced wound healing speed, even slower than in control cells without TGF-β challenge, indicating that the HAPLN1 protein reduces CRC epithelial cell growth.

### HAPLN1 Regulates Collagen *via* the TGF-β Signaling Pathway in CRC

**Figure 6:**
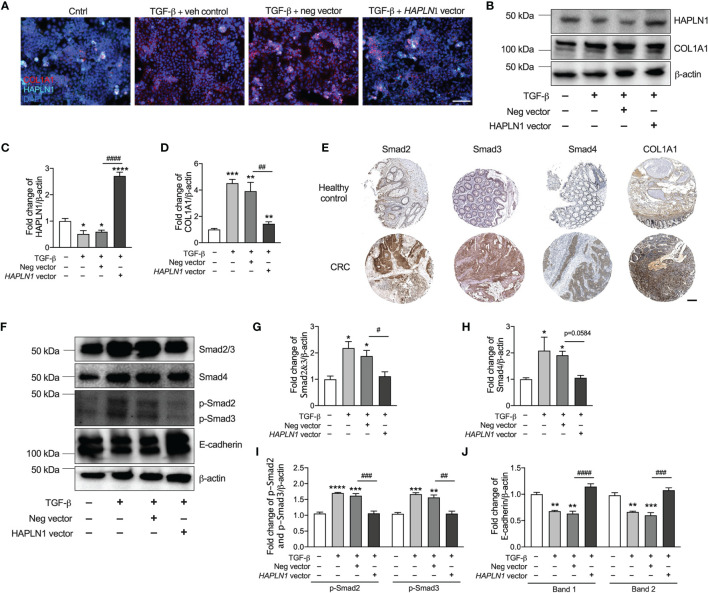
HAPLN1 regulates collagen *via* the TGF-β signaling pathway and E-cadherin to control tumor growth in colorectal cancer (CRC). Human CRC epithelial (Caco-2) cells were transfected with *HAPLN1* overexpression or negative control plasmid, and vehicle controls received media. Cells were then challenged with human recombinant TGF-β protein. **(A)** Cells were stained with HAPLN1 and COL1A1 fluorescent antibody after 24 h of TGF-β and assessed by immunofluorescence (scale bar = 100 μm). **(B)** HAPLN1 and COL1A1 proteins were assessed in cell lysates by immunoblot. Fold change of densitometry of HAPLN1 **(C)** and COL1A1 **(D)** was normalized to β-actin. **(E)** Smad2, Smad3, Smad4, and COL1A1 proteins in human colon healthy controls and tumors from CRC patients by immunohistochemistry in the Pathology Atlas database (scale bar = 200 μm). **(F)** Smad2/3, Smad4, p-Smad2/3, and E-cadherin proteins were assessed in cell lysates by immunoblot. Fold change of densitometry of Smad2/3 **(G)**, Smad4 **(H)**, p-Smad2/3 **(I)**, and E-cadherin **(J)** was normalized to β-actin. *n* = 4. Results are mean ± SEM. *P < 0.05, **P < 0.01, ***P < 0.001, ****P < 0.0001 compared with control cells receiving media. ^#^ *P* < 0.05, ^##^ *P* < 0.01, ^###^ *P* < 0.001, ^####^ *P* < 0.0001 compared with cells transfected with negative control plasmid.

To assess the mechanisms of how HAPLN1 regulates CRC epithelial cell growth, we transfected these cells with *HAPLN1* overexpression plasmid with and without TGF-β for 24 h. TGF-β significantly reduced the numbers of HAPLN1-positive cells but increased collagen proteins (COL1A1) in those cells (**Figure 6A**). *HAPLN1* overexpression restored the numbers of HAPLN1-positive cells but reduced collagen deposition after TGF-β challenge.

To confirm that HAPLN1 regulates collagen in CRC, we also collected cell lysates 24 h after *HAPLN1* overexpression with or without 24 h TGF-β challenge. TGF-β reduced the HAPLN1 protein in CRC epithelial cells, and *HAPLN1* overexpression increased the HAPLN1 proteins after TGF-β challenge compared with control cells (**Figures 6B, C** and **Supplementary Figure S2A**). *HAPLN1* overexpressing cells also had reduced collagen after TGF-β challenge (**Figure 6D**).

Given that reduced HAPLN1 was associated with increased collagen in CRC, we investigated the mechanism of how HAPLN1 regulates collagen in CRC. Previous studies showed that the TGF-β signaling pathway regulates collagen deposition (10, 22); thus, we assessed the key downstream proteins (Smad2, Smad3, Smad4, and COL1A1) in this signaling pathway in normal colon tissue and tumors from CRC patients. Smad2, Smad3, Smad4, and COL1A1 proteins were strongly expressed in tumor tissues from CRC patients compared with normal colon controls (**Figure 6E**). We also measured Smad2/3, p-Smad2/3, and Smad4 proteins in an *in-vitro* model of CRC. We found that Smad2/3, p-Smad2/3 and Smad4 proteins were significantly increased in CRC epithelial cells after TGF-β challenge, but were decreased with *HAPLN1* over-expression (**Figures 6E–I** and **Supplementary Figure S2B**).

To further understand how HAPLN1 regulates CRC growth, we assessed E-cadherin protein in our *in-vitro* model of CRC. E-cadherin is an important molecule involved in cell adhesion and tumor development (29). TGF-β challenge increased E-cadherin protein levels in CRC epithelial cells; however, the levels were significantly decreased with *HAPLN1* overexpression compared with cells transfected with the negative control plasmid (**Figure 6J**).

## Discussion

HAPLN1 is an ECM protein that contributes to cell proliferation, cell–cell interactions, and tumor development. In this study, we found that the *HAPLN1* gene and proteins are decreased in tumors from CRC patients compared with colon healthy controls and normal colon regions of CRC patients. We also show that decreases in HAPLN1 proteins are associated with increased COL1A1 protein levels in CRC epithelial cells after TGF-β challenge and control tumor growth. A potential mechanism is that HAPLN1 regulates tumor cell proliferation *via* the TGF-β (Smad2/3, p-Smad2/3, and Smad4) signaling pathway. HAPLN1 also mediates E-cadherin to regulate tumor cell attachment in CRC. However, *HAPLN1* overexpression restored COL1A1, TGF-β signaling, and E-cadherin proteins in CRC epithelial cells.

Studies have shown that *HAPLN1* mRNA is downregulated in chondrocytes in endoplasmic reticulum stress-induced cartilage damage (30). *HAPLN1* expression is significantly reduced by interleukin-1 alpha stimulation and further results in decreased cell proliferation in cartilage injury (31). Previous studies showed that *HAPLN1* mRNA was decreased in brain cells 24 h after TGF-β-induced brain damage (11). These studies indicate that HAPLN1 is associated with cell growth, but HAPLN1 levels vary in different cancers. *HAPLN1* gene expression is increased in lung tissues from patients with pleural mesothelioma compared with healthy controls (32), while some other studies showed HAPLN1 levels decreased during aging, and this is potentially involved in cancer invasion (6). The decrease in HAPLN1 protein results in the disruption of the vascular basement membrane and induces vessel permeability, and this enhances melanoma metastasis (6). To our knowledge, only one other study has assessed the *HAPLN1* gene in a small cohort (*n* = 15) of CRC patients and it was found to be elevated in CRC (12). However, previous studies indicated that *HAPLN1* expression may be altered during colorectal carcinogenesis (33). In our study, we observed a decreased gene expression of *HAPLN1* in CRC patients compared with healthy controls and normal tissue adjacent to the tumor. The decreased in HAPLN1 protein has also been confirmed in CRC patients by histological analysis in a larger cohort (*n* = 59). The HAPLN1 protein is further decreased in severe CRC patients, indicating a correlation between HAPLN1 and CRC severity. Low levels of *HAPLN1* gene in the colon are associated with a lower survival rate of CRC patients, indicating that factor downregulation of HAPLN1 may drive CRC development. We have also shown that epithelial, stromal, and endothelial cells and myofibroblasts express *HAPLN1* gene in the colon by RNA-sequencing, but only epithelial cells are associated with tumor formation in CRC.

Abnormal deposition of ECM leads to tissue stiffening and is associated with the tumor microenvironment in CRC (34). Collagen is the most abundant component of ECM protein and increased collagen deposition results in tissue remodeling and tumor formation (10). We previously showed that collagen (COL1A1 and COL3A1) mRNAs are increased in tumor tissues from CRC patients compared with normal tissue adjacent to the tumor (26). In this study, we show that COL1A1 protein is significantly increased in CRC epithelial cells after TGF-β challenge. We also show that HAPLN1 correlated with collagen in CRC, indicating that HAPLN1 may regulate collagen in the tumor microenvironment. Increased collagen deposition in tissues results in cancer cell proliferation and tumor growth (35). However, increased collagen is associated with decreased HAPLN1 in CRC epithelial cells after TGF-β challenge. We also observed an increase of HAPLN1 protein in CRC epithelial cells after 24 h of *HAPLN1* overexpression compared with earlier time points. This leads to a decrease of collagen in the cancer cells, indicating that HAPLN1 may regulate collagen formation in CRC.

TGF-β is an important cytokine in the regulation of cell proliferation, differentiation, adhesion, and migration in many cell types (36). It is a key regulator of ECM products and the microenvironment in cancers (36). The TGF-β signaling pathway is involved in cancer development and tumor growth in CRC, and Smad family members are important downstream molecules in the TGF-β signaling pathway (37). Studies show that *Smad2* knockout mice are embryonic lethal (38). Smad3 protects against the development of colorectal tumors, and specifically, knockout of exon 2 in *Smad3* promotes metastasis of large bowel cancer (39). However, knockout of exon 8 in *Smad3* results in autoimmunity with abnormally activated T cells, and this induces colon inflammation and adenocarcinomas (40). *Smad4* knockout mice are prone to embryonic lethality due to defects in gastrulation (41). Depletion of Smad4 from T cells in mice leads to epithelial carcinomas in the gut (42). We show that TGF-β stimulation increases both Smad2/3 and p-Smad2/3 proteins in CRC epithelial cells, and this confirmed the findings of a previous study (43). This demonstrates that the balance of Smad proteins is critical in tumor formation in CRC. We have shown that *HAPLN1* overexpression in cancer cells restored p-Smad2/3 to control levels, indicating that HAPLN1 may regulate the TGF-β signaling pathway.

Changes in the tissue microenvironment contribute to cell–cell adhesion, and loss of adhesion is a key mechanism of cancer invasion and progression (44). E-cadherin is a key adhesion protein that promotes cell–cell adhesion and maintains epithelial morphology (45). Studies have identified that low levels of E-cadherin expression are associated with colon cancer invasiveness (44). The expression of E-cadherin in cancer patients with metastasis is lower than that in normal tissue adjacent to the tumor (45). Patients with cancer had decreased E-cadherin expression and this led to a lower survival time (44). This indicates that E-cadherin is associated with tumor differentiation, invasion, lymph node metastasis, and severity of cancer (46). In this study, we have shown that TGF-β challenge reduces E-cadherin protein in CRC cells, and this confirmed previous observations (47). However, overexpression of *HAPLN1* in CRC cells increased E-cadherin proteins after TGF-β stimulation, indicating that HAPLN1 regulates E-cadherin and reduces tumor cell growth.

**Figure 7:**
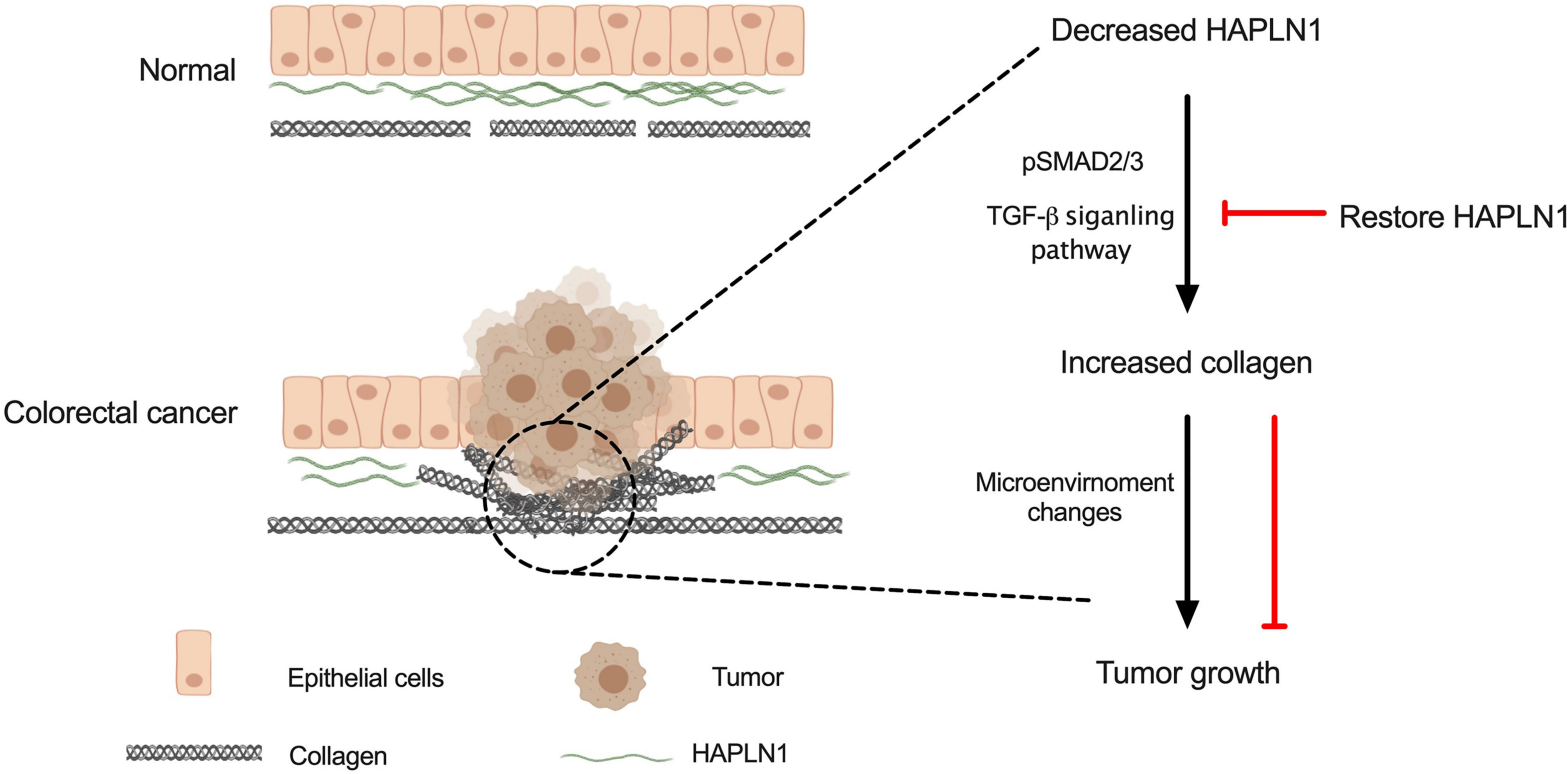
Schematic of HAPLN1 regulation of collagen and tumor growth. Decreased HAPLN1 expression leads to increased collagen deposition *via* pSmad2/3 in the TGF-β signaling pathway, resulting in tumor growth. Restoring HAPLN1 levels can reduce collagen and inhibit tumor growth.

Taken together, our data show that low levels of HAPLN1 induces collagen deposition in the colon *via* p-Smad2/3 involved in the TGF-β signaling pathway. Increased collagen leads to cell microenvironment changes and tumor cell proliferation, migration, and invasion in CRC. *HAPLN1* overexpression restored HAPLN1 protein and reduced cancer cell growth (**Figure 7**). This provides a new therapeutic approach by restoring HAPLN1 in CRC patients.

## Data Availability Statement

The datasets presented in this study can be found in online repositories. The names of the repository/repositories and accession number(s) can be found below: https://www.ncbi.nlm.nih.gov/geo/, GSE128449 and https://www.ncbi.nlm.nih.gov/geo/, GSE110224.

## Ethics Statement

The studies involving human participants were reviewed and approved by Bengbu Committee of Bengbu Medical College. Written informed consent for participation was not required for this study in accordance with the national legislation and the institutional requirements. Written informed consent was obtained from the individual(s) for the publication of any potentially identifiable images or data included in this article.

## Author Contributions

YW performed the *in-vitro* experiments and data analysis. XX performed the statistical analysis and revised the manuscript. MG and JM assisted in the experimentation. YZ, KD, PH, and JX revised the manuscript. GL designed the study, performed some *in-vitro* experiments, and prepared the manuscript. All authors contributed to the article and approved the submitted version.

## Funding

This work was supported by TSANZ and the Australian Lung Foundation (GL). XX is supported by the Australia National Heart Foundation post-doctoral fellowship and the University of New South Wales Scientia Program. PH is funded by a fellowship and grants from the National Health and Medical Research Council (NHMRC) of Australia (1175134), University of Technology Sydney (UTS), and Cancer Council of NSW (1099119, 1157073).

## Conflict of Interest

Author YW was employed by Hangzhou Xunyao Biotechnology Pty. Ltd.

The remaining authors declare that the research was conducted in the absence of any commercial or financial relationships that could be construed as a potential conflict of interest.

## Publisher’s Note

All claims expressed in this article are solely those of the authors and do not necessarily represent those of their affiliated organizations, or those of the publisher, the editors and the reviewers. Any product that may be evaluated in this article, or claim that may be made by its manufacturer, is not guaranteed or endorsed by the publisher.
