# Supplementary material for: Loss of Hyaluronan and Proteoglycan Link Protein-1 Induces Tumorigenesis in Colorectal Cancer
Source: Front Oncol. 2021 Dec 13;11:754240. doi: 10.3389/fonc.2021.754240 (PMC8710468; doi:10.3389/fonc.2021.754240)
Supplement: Supplementary file 1 [file DataSheet_1.docx]

**Supplementary materials**

**Loss of Hyaluronan and Proteoglycan Link Protein-1 Induces** **Tumorigenesis in Colorectal Cancer**

**Yao Wang^1,2^**^†^**, Xiaoyue Xu^3^**^†^**, Jacqueline E Marshall^4,5^, Muxue Gong^6^, Yang Zhao^7^, Kamal Dua^4,8^, Philip M Hansbro^4,5^, Jincheng Xu^9,10^, Gang Liu^4,5^**^*^

^1^College of Biology and Food Engineering, Anyang Institute of Technology, Anyang, Henan, China

^2^Hangzhou Xunyao Biotechnology Pty. Ltd. Hangzhou, Zhejiang. China

^3^School of Population Health, University of New South Wales, Sydney, New South Wales, Australia

^4^Centre for Inflammation, Centenary Institute Sydney, NSW, Australia.

^5^School of Life Sciences, Faculty of Science, University of Technology Sydney, NSW Australia

^6^School of Clinical Medicine, Bengbu Medicine College, Bengbu, Anhui, China

^7^Department of Biochemistry and Molecular Biology, School of Medicine, Nanjing University of Chinese Medicine, Nanjing, Jiangsu, China

^8^Discipline of Pharmacy, Graduate School of Health, University of Technology Sydney, Ultimo, NSW, Australia

^9^Stomatology Department, The First Affiliated Hospital of Bengbu Medical College, Bengbu, Anhui, China

^10^School of Dental Medicine, Bengbu Medical College, Bengbu, Anhui, China

^†^Contributed equally

***Correspondence:**

Dr Gang Liu

Address: Centenary Institute, Level 3, building 93, Royal Prince Alfred Hospital, Missenden Road, Camperdown NSW 2050 Australia.

E-mail: gang.liu@uts.edu.au

**Supplementary figure and figure legends**

**
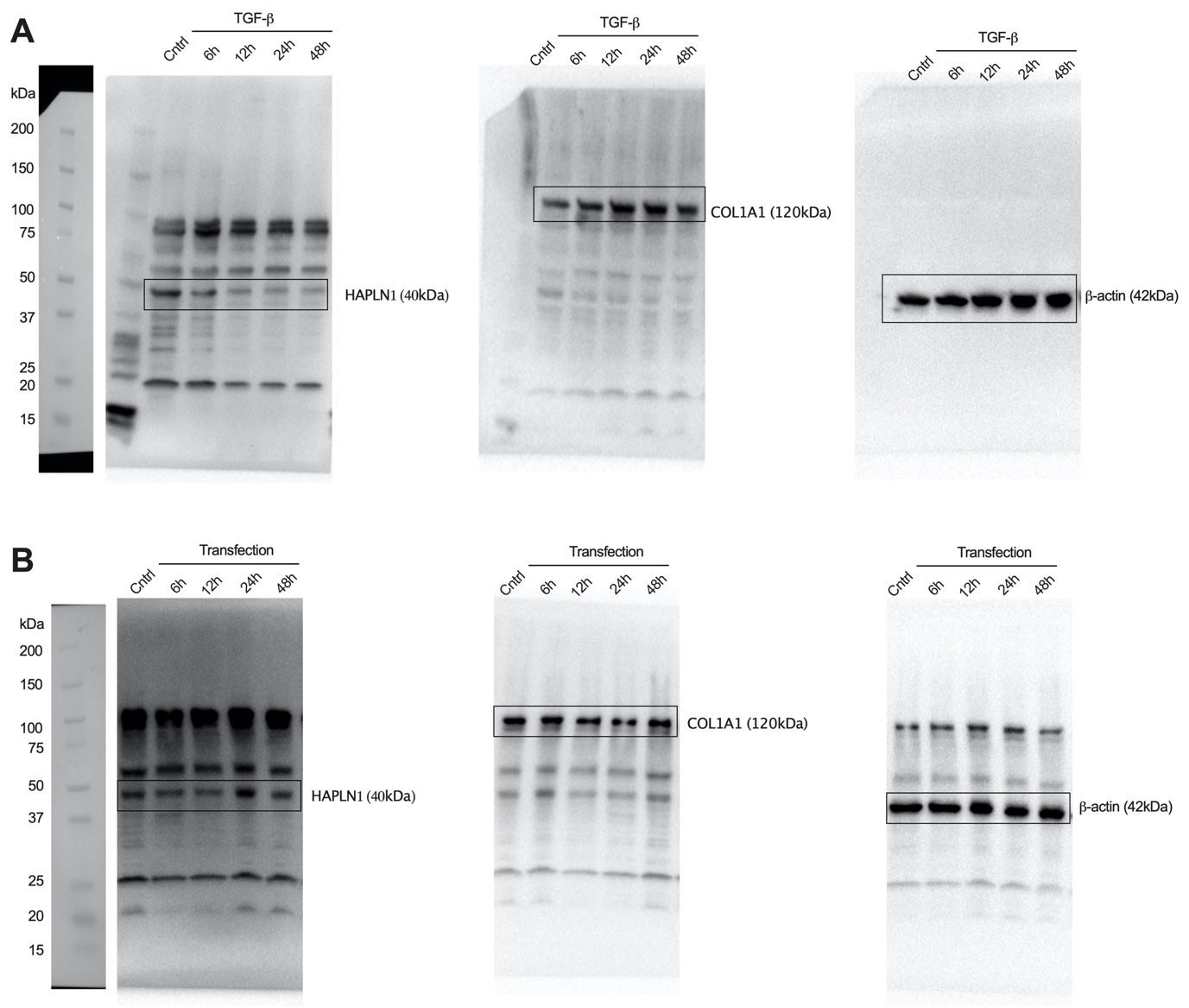
Figure S1. Full length immunoblots of HAPLN1, COL1A1 and -actin in Fig. 3A and Fig. 4C**. **A** Human colorectal cancer cells (Cacao-2) were treated with recombinant TGF-β protein, and controls cells received media. HAPLN1 and COL1A1 were assessed in cell lysates during time course (6, 12, 24 and 48 h) of TGF-β protein stimulation by immunoblotin, and rectangle indicates the cropped representative image in **Figure. 3A**. **B** *HAPLN1* over-expression plasmids were transfected into Cacao-2. HAPLN1 and COL1A1 proteins were assessed in cell lysates after time course of *HAPLN1* over-expression plasmid transfection (0, 6, 12, 24 and 48 h) by immunoblot, and rectangle indicates the cropped representative image in **Figure. 4C**.

**
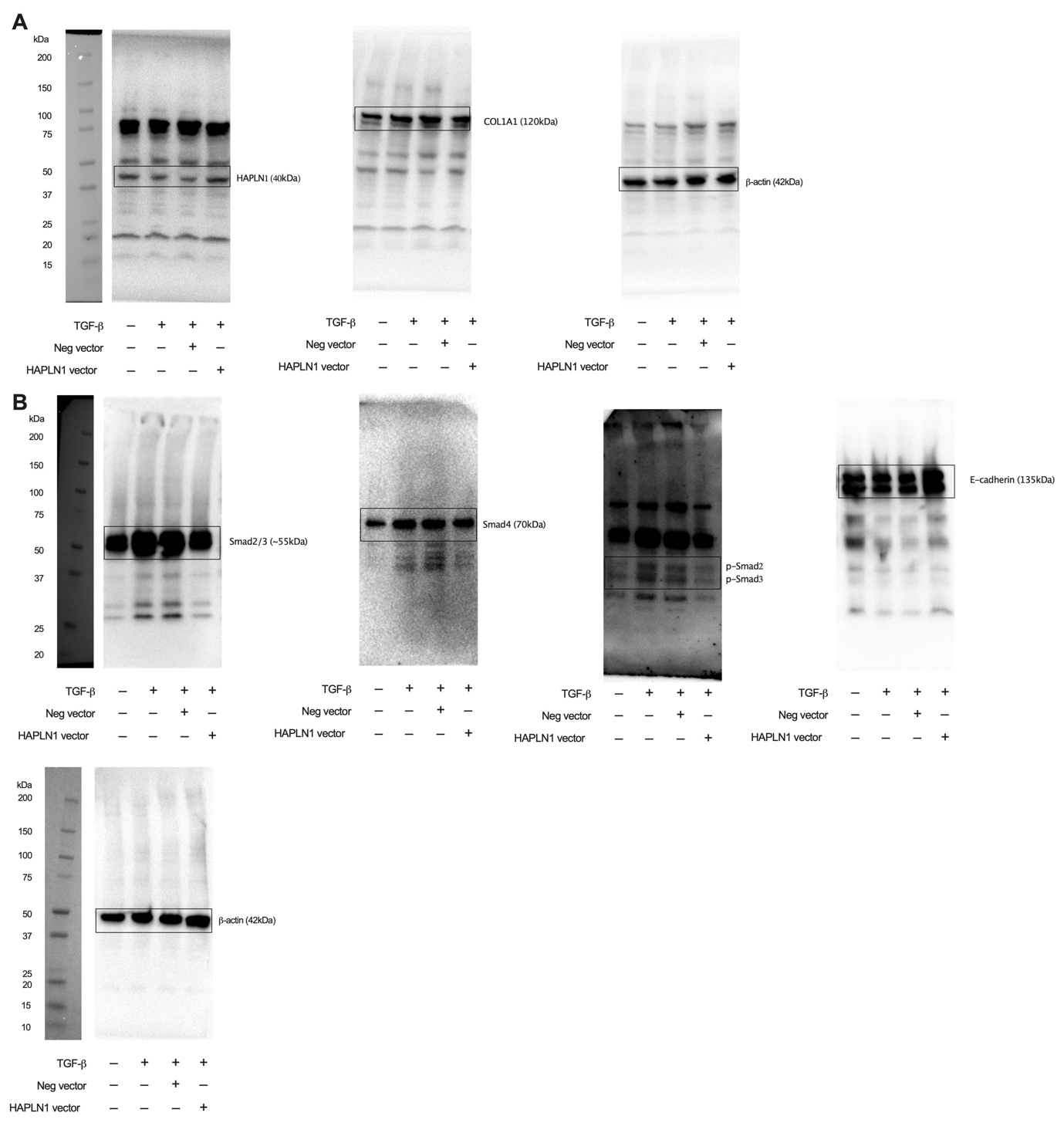
**

**Figure S2. Full length immunoblots of HAPLN1, COL1A1, Smad2/3, Smad4 and E-cadherin in Figure. 6**. Human colorectal cancer cells (Caco-2) were received *HAPLN1* over-expression plasmid or negative control plasmid, and vehicle control group was received cell media. The cells were then stimulated with human recombinant TGF-β protein. **A** HAPLN1 and COL1A1 proteins were assessed in cell lysates by immunoblot, and rectangle indicates the cropped representative image in **Figure. 6B**. **B** Smad2/3, Smad4, p-Smad2, p-Smad3 and E-cadherin proteins were assessed in cell lysates by immunoblot. Rectangle indicates the cropped representative image in **Figure. 6F**.
